# Feasibility and effects of enhanced recovery vs. conventional care after emergency colon surgery for patients with left colon perforation

**DOI:** 10.1038/s41598-020-64242-7

**Published:** 2020-04-30

**Authors:** X. Viñas, E. Macarulla, C. Brugiotti, J. M. Ramirez, A. Pedregosa, S. Sanchez, J. Camps, A. Arroyo

**Affiliations:** 1 Hospital General de Igualada. Servicio de Cirugía General y del Aparato Digestivo. Igualada, 08700 Barcelona, Spain; 2 Hospital General de Inca. Servicio de Cirugía General y del Aparato Digestivo. Inca 0730, Mallorca, Spain; 3 Hospital General Universitario de Elche. Servicio de Cirugía General y del Aparato Digestivo. Elche 03203, Alicante, Spain; 4 Hospital Clínico Universitario Lozano Blesa. Zaragoza. Servicio de Cirugía General y del Aparato Digestivo, Zaragoza, 50009 Spain; 5 Hospital General de Igualada. Servicio de Anestesiología y Reanimación. Igualada, 08700 Barcelona, Spain

**Keywords:** Gastrointestinal diseases, Gastrointestinal diseases

## Abstract

The impact of an enhanced recovery after surgery (ERAS) programme in emergency colorectal surgery has not yet been reported. The objective of this study was to evaluate the feasibility and the results of patients included in an ERAS protocol following emergency colon surgery for left colon perforation. For this purpose, patients with a low to moderate risk of mortality, according to a Peritonitis Severity Score (PSS), and treated with an ERAS protocol (ERAS group) after emergency surgery for left colon perforation were compared for a period of 40 months (March 2014–June 2017) with a control group of patients treated with conventional care (CC group) during the 38 months prior to implementation of the new ERAS protocol (January 2011–February 2014). The main endpoint was 90-day postoperative morbidity according to the Clavien–Dindo classification. Secondary endpoints included length of postoperative hospital stay, 90-day readmission rate, protocol compliance and mortality. Fifty patients were included in the study, 29 in the ERAS group and 21 in the CC group. There were no significant differences between the groups in the demographic data or in the operative characteristics. A reduction in the incidence of postoperative complications (20.7% vs. 38%; p > 0.05) and in the postoperative hospital stay (7.7 + /- 3.85 vs. 10.9 + /- 5.6 days; p = 0.009) were observed in the ERAS group. The 90-day readmission rate did not differ significantly between the two groups (2 vs. 1). No 90-day mortality was observed in either group. The ERAS group showed better results than the CC group in protocol compliance. We conclude that ERAS protocols are feasible and help to reduce morbidity and length of hospital stay without adversely affecting the rate of readmission or mortality.

## Introduction

Enhanced recovery after surgery (ERAS) programmes in elective colorectal surgery are widespread and broadly accepted. The results of several multicentre studies^1,2^, controlled and randomised trials^3^, and corresponding meta-analyses^4,5^ have shown significant reductions in morbidity and postoperative hospital stays when applying these programmes compared to outcomes when applying conventional perioperative care in patients undergoing elective colorectal surgery.

As of 2008, the interdisciplinary GERM^1^ (Spanish Multimodal Rehabilitation Group) team has applied ERAS guidelines to patients undergoing surgical procedures for colon resection, with extensions to other elective gastrointestinal surgical procedures. The Spanish Ministry of Health recently published^6^ the clinical pathway for enhanced recovery in elective abdominal surgery (RICA) with the aim of homogenising and standardising all related procedures and processes.

However, things are different in emergency situations. Due to the complexity of individual patients and their management, application of the ERAS guidelines to patients undergoing emergency colon surgery has not been widespread. Some authors^7–11^ have reported that the preliminary results of these programmes demonstrate their viability and effectiveness for this type of surgical patient.

One condition requiring emergency surgery is perforation of the left colon, which is often secondary to acute diverticulitis, with other less frequent causes including tumour, iatrogenic sources, ischaemia and perforation by foreign body^12^. These patients are vulnerable to sepsis secondary to perforation and subsequent peritonitis. The severity of a patient’s condition and the operative risk (which contribute to the patient’s prognosis and selection of the appropriate surgical procedure) vary. Prognostic scales based on objective results, such as the PSS classification (Peritonitis Severity Score)^13^, facilitate appropriate management of each situation and patient.

Based on previous experience gained since 2009 and on the management of a multimodal rehabilitation guide applicable to patients submitted to elective colorectal surgery^1,2^, in 2014 GERM proposed new ERAS guidelines for the management of patients undergoing emergency colon surgery. To the best of our knowledge, this is the first published study on the applicability and results of a specific ERAS protocol in the management of patients with left colon perforation requiring emergency colon surgery.

## Methods

The study was designed as a two-centre prospective study controlled with a retrospective group.

A consecutive series of patients (the ERAS group) from the General Hospital of Igualada (Barcelona) and the University Hospital of Elche (Alicante)—both of which are GERM members—who underwent emergency surgery with a diagnosis of left colon perforation received perioperative care according to the ERAS protocol between March 2014 and June 2017. This new specific protocol adapted by the GERM team is shown in Tables 1 and 2.

**Table 1:** Preoperative and intraoperative ERAS protocol for emergency colon surgery.

Preoperative
• Antibiotic and thromboembolic prophylaxis (as per the hospital protocol)
• Blood glucose control
• C-reactive protein
• Stoma marking
• No anaesthetic premedication
Intraoperative
Anaesthetic procedure
• Nausea and vomiting prophylaxis: dexamethasone (5 mg) and ondansetron (4 mg)
• Epidural catheter for analgesia (T6−T10 according to incision): levobupivacaine 0.125−0.0675%.
• Basic ASA.
• Non-invasive blood pressure, pulse oximetry, heart rate.
• Hypothermia prevention (thermal blanket 37 °C).
• Optimisation of fluid therapy with Doppler monitoring whenever possible.
• Intravenous fluid balance: fluid therapy with continuous infusion of balanced solution (3.5 ml/kg/h for laparoscopic surgery and 7 ml/kg/h for laparotomy).
• Invasive monitoring if necessary (central line, arterial catheter) to avoid central intravenous catheters.
• Maintenance of anaesthesia with total intravenous anaesthesia.
• Infiltration of local anaesthetic into the incision and trocar sites.
• Avoid opiates (use short-acting opioids if needed).
• Maintain blood haemoglobin levels above 8 g/dL.
Surgical procedure
• Laparoscopy or laparotomy.
• Avoid abdominal drains.
• Nasogastric aspiration, if present, will be removed before extubation or within <12 postoperative hours.

**Table 2:** Postoperative ERAS programme for emergency colon surgery.

PO Day	Diet	Medication	Mobilisation	Removal of tubes	Blood Tests
0	Start fluid intake after 20 to 24 PO hours	Analgesia Paracetamol 1 g IV/8 h. (alternate) Dexketoprofen 50 mg IV/8 h. (alternate) Omeprazole 20 mg IV/24 h Antibiotics as per protocol Enoxaparin 40 mg Sc/24 h	Start sedestation		
1	Progressive diet according to tolerance	Same	Sedestation Start ambulation		
2	• Progressive diet according to tolerance • Stop IV fluid therapy if tolerated. • Maintain heparinised IV	If diet is tolerated: • Analgesia: Paracetamol 1 g oral/8 h Ibuprofen 600 mg oral/8 h (alternate) • Omeprazole 20 mg oral/24 h • Antibiotics as per protocol • Enoxaparin 40 mg Sc/24 h	Equal	• Removal of epidural analgesia • Removal of bladder catheter • Removal of abdominal drainage	
3	Basal diet	Same	Same		With CRP
4/5	Basal diet	Same	Same		CPR (5 days)
6	Basal diet	Discharge criteria: No complications, controlled pain, complete ambulation, patient acceptance.			

The matched control group received conventional perioperative care (the CC group). Controls were identified by searching an electronic database for consecutive patients who underwent surgery in our hospitals between January 2011 and February 2014 (before introduction of the ERAS programme) and met the inclusion and exclusion criteria for ERAS.

The inclusion criteria were patients aged over 18 years with a low to moderate risk of mortality according to a Peritonitis Severity Score (PSS; between 6 and 11 points). The exclusion criteria were patients aged less than 18 years and those with a high risk of mortality according to the PSS (12 points or higher).

The main endpoint was 90-day postoperative morbidity according to the Clavien–Dindo classification. The secondary endpoints were length of postoperative hospital stay, 90-day readmission rate, protocol compliance and mortality.

The preoperative variables analysed were sex, age, perforation aetiology, antibiotic prophylaxis, glycaemic control, CPR determination, stoma marking, and premedication. The intraoperative variables analysed were ASA (American Society of Anaesthesiologists) classification, nausea and vomiting prophylaxis, epidural analgesia, hypothermia prevention, fluid optimisation (monitoring), opiate use, surgical procedure performed, access to the abdominal cavity (laparotomy or laparoscopy) and PSS. The postoperative variables analysed were removal of the nasogastric tube (NGT) before 12 hours, active mobilisation of the patient, oral intake before 24 hours, removal of abdominal drainage tubes before 48 hours, removal of intravenous and urinary catheters at 48 hours, CPR levels, complications, prognosis according to the Clavien–Dindo classification^14^, mortality, length of hospital stay and postoperative readmissions within 90 days. Protocol compliance in the ERAS group was also analysed. The data were recorded using Microsoft Office ® Excel software, and the analysis was conducted using SPSS ® software.

Independence between qualitative variables was checked using the chi-square test or Fisher’s exact test, which was used when the chi-square test assumptions were not met. Independence between quantitative variables was checked using Student’s t-test or Mann–Whitney U test, which was used when normality assumptions were not met.

This study was carried out in accordance with the Helsinki Declaration and approved by the Ethics Committee of the Elche University Hospital. Patients in the ERAS group gave written informed consent before the study. Informed consent of the control group was waived by the approving ethics committee.

## Results

A total of 50 patients were included in the study: 29 in the ERAS Group and 21 in the CC group.

No significant differences between the ERAS group and the CC Group were observed in the patients’ demographic data or in the operative characteristics (sex, age, aetiology of colonic perforation, surgical procedure performed, access to the abdominal cavity (laparotomy or laparoscopy) and PSS) (see Table 3).

**Table 3:** Demographic data and operative characteristics for patients in the ERAS group versus the conventional care (CC) group.

	ERAS group (n = 29)	CC group (n = 21)	Statistical significance p < 0.05
Sex (M = male, F = female)	21 M/8 F	17 M/4 F	N/S
Age (mean ± standard deviation)	54 ± 3,08	58 ± 2,87	N/S
Aetiology			
• Acute diverticulitis (AD)	24	17	N/S
• Neoplasm (N)	2	1	N/S
• Iatrogenic (Y)	2	3	N/S
• Ischaemia (I)	1	0	N/S
Surgical intervention			
• Resection and anastomosis (RA)	11	10	N/S
• Hartmann (HP)	13	7	N/S
• Lavage and drainage (LD)	3	2	N/S
• Simple suture (SS)	2	2	N/S
PSS score (mean ± standard deviation)	7.7 ± 2.5		

No significant differences in compliance were found for four of the preoperative items analysed (antibiotic prophylaxis, thromboembolic prophylaxis, glycaemic control, and premedication), although significant differences were found for C-reactive protein determination (ERAS 24/29 vs. CC 7/21) and stoma marking (ERAS 10/29 vs. CC 0/21; p < 0.05) in the ERAS group (see Table 4).

**Table 4:** Perioperative items (ERAS group vs CC group) and their compliance in the ERAS group.

Items	ERAS group (n = 29)	ERAS group compliance (%)	CC group (n = 21)	CC group compliance (%)	Statistical significance p < 0.05
**Preoperative items**					
Antibiotic prophylaxis	29/29	100%	21/21	100%	N/S
Thromboembolic prophylaxis	29/29	100%	21/21	100%	N/S
Glycaemia control	29/29	100%	21/21	100%	N/S
Determination of C-reactive protein	24/29	82.7%	7/21	33%	<0.01
Stoma marking	10/29	34.4%	0/21	0%	0.003
No anaesthesic premedication	29/29	100%	21/21	100%	N/S
**Intraoperative items**					
Nausea and vomiting prophylaxis	18/29	62%	6/21	28.5%	0.04
Epidural analgesia	11/29	38%	7/21	33%	N/S
Hypothermia prevention	6/29	20.6%	2/21	9.52%	N/S
Fluid optimisation (monitoring)	11/29	38%	0/21	0%	0.001
No opiates	19/29	65.5%	4/21	19.5%	0.003
Laparoscopic colon resection	4/29	13.7%	0/21	0%	N/S
Lavage + drainage via laparoscopy	3/29	10.3%	2/21	9.52%	N/S
Total laparoscopic access	7/29	24%	2/21	9.52%	N/S
No abdominal drains	3/29	10.3%	1/21	4.76%	N/S
**Postoperative items**					
NGT removal within 12 PO hours	23/29	79.3%	0/21	0%	<0.001
Start mobilisation within 24 PO hours	23/29	79.3%	0/21	0%	<0.001
Start oral intake within 24 PO hours	23/29	79.3%	0/21	0%	<0.001
Remove abdominal drains 2^nd^ day PO	11/26	42.3%	1/21	4.76%	0.009
Remove IV therapy 2^nd^ day PO	7/29	24%	0/21	0%	<0.001
Remove urinary catheter 2^nd^ day PO	23/29	79.3%	0/21	0%	<0.001
CRP blood levels 3^rd^ day	24/29	82.7%	5/21	23.8%	<0.01

No significant differences in compliance were found for six of the intra-operative items analysed (epidural analgesia, hypothermia prevention, laparoscopic colon resection, laparoscopic lavage + drainage, overall laparoscopy access and abdominal drains), although significant differences were found for nausea and vomiting prophylaxis (ERAS 18/29 vs. CC 6/21), monitored fluid optimisation (ERAS 11/29 vs. CC 0/21) and opiate use (ERAS 19/29 vs. CC 4/21; p < 0.05) in the ERAS group (see Table 4).

Significant differences were found for all postoperative items in the ERAS group, including removal of the nasogastric tube before 12 postoperative hours (ERAS 23/29 vs. CC 0/2), mobilisation before 24 postoperative hours (ERAS 23/29 vs. CC 0/21), oral intake before 24 postoperative hours (ERAS 26/29 vs. CC 0/2), abdominal drain removal before 48 postoperative hours (ERAS 11/26 vs. CC 1/20), intravenous catheter removal on the second postoperative day (ERAS 7/29 vs. CC 0/21), urinary catheter removal on the second postoperative day (ERAS 23/29 vs. CC 0/21), and CRP levels on the third postoperative day (ERAS 24/29 vs. CC 5/21; p < 0.05) (see Table 4).

No significant differences were found for overall morbidity and mortality or for the readmission rate in either group, although a significantly reduced length of hospital stay was observed in the ERAS group (ERAS 7.7 ± 3.85 vs. 10.9 ± 5.6 days; p = 0.009) (see Table 5).

**Table 5:** Postoperative morbidity/mortality, length hospital stay, readmissions.

Morbidity Clavien–Dindo Classification	ERAS group (n = 29)	CC group (n = 21)	Statistical significance p < 0.05
• I	2	3	N/S
• II	0	1	N/S
• IIIa	3	2	N/S
• IIIb	1	1	N/S
• IVa	0	1	N/S
Overall morbidity	6 (20.7%)	8 (38%)	N/S
Mortality	0	0	N/S
Readmissions	2 (6.8%)	1 (4.7%)	N/S
Length hospital stay	7 (6-8)	9 (1-12)	0.009

## Discussion

The results of implementing an ERAS programme in elective colon surgery showed a positive impact^1–5^, thus supporting the national model developed in Spain or the IMPRICA (Spanish ERAS National implementation project) using a multi-centre and interdisciplinary strategy^6^. Our current study is the first to report the results of patients treated with an ERAS protocol compared with those of a group of patients treated with conventional care following emergency colon surgery for left colon perforation according to the PSS classification. This study demonstrates that a specific ERAS protocol has a positive impact on patient outcomes, thereby accelerating patient recovery without compromising patient safety.

Few studies on the implementation of ERAS guidelines in emergency colon surgery are currently available. Verheijen *et al*.^7^ analysed the feasibility of ERAS in various patient groups based on the outcomes of 348 patients who underwent colon surgery and were managed using an ERAS programme, including 41 (12%) patients who underwent emergency surgery. The only significant differences after multivariate analysis were a decreased reoperation rate (8% vs. 26%) and reduced length of hospital stay (7 vs. 14 days) in the elective surgery group. Readmission and the anastomotic leak rate were similar in both groups. However, the study did not use a specific ERAS protocol and did not define the indications for emergency surgery.

Similarly, Loshiriwat *et al*.^8^ reported a comparative study of 20 patients with obstructive cancer colon who required emergency colectomy and were treated using an ERAS programme and 40 patients who were treated with conventional management. Roulin *et al*.^9^ reported another comparative study on the applicability of an ERAS programme in 28 patients (nine with colon perforation) after emergency colon surgery compared with 63 patients who underwent elective colon surgery. ERAS programmes were found to be feasible in emergency surgery and did not increase morbidity or postoperative mortality in either study.

In 2017, a comparative study by Shida *et al*.^10^ included 122 consecutive patients undergoing emergency surgery for obstructive colon neoplasia, 42 who were treated with conventional management and 80 who were treated using a modified ERAS programme. The results demonstrated reduced length of hospital stay, with no increase in postoperative morbidity or mortality.

A systematic review by Padararu *et al*.^15^ involved a search for ERAS protocols in emergency cases in the PubMed and Cochrane databases. These authors selected for analysis four cohort studies^7–9,11^, including three that were specific for emergency colon surgery^7–9^ and one by Wiseley *et al*.^11^ in which 370 patients undergoing emergency abdominal surgery and 151 patients (43%) undergoing colectomy (83 patients submitted to right colectomy, 21 to left colectomy and 53 to Hartmann’s intervention) were treated using ERAS protocols. In this latter paper, a fifth randomised study^16^ of patients undergoing emergency surgery for perforated duodenal ulcers was reported. Padararu^15^ concluded that the application of ERAS programmes to patients undergoing emergency gastrointestinal surgery improves their management without increasing morbidity or mortality.

Unlike the studies in the above-mentioned systematic review^7–9,11^, our study reported the results of a specific ERAS protocol for the management of patients undergoing emergency surgery of the same condition (perforation of the left colon) and aetiology (acute diverticulitis was the most frequent cause of perforation). In addition, all patients were homogeneous in terms of demographic data, surgical procedure and the severity of left colon perforation according to the Peritonitis Severity Score (PSS). Middle age, absence of aetiology of cancer-related perforation and a low to moderate risk of mortality according to the PSS are very important items for the selection of patients who are likely to benefit from an ERAS protocol.

Regarding the main endpoint of our study, in other words, impact of the new ERAS protocol on the postoperative course at 90 days, the new management showed significantly lower morbidity rates (raw numbers and Clavien–Dindo classification)^14^, without adding cases of mortality or readmission. The ERAS group also exhibited a significantly shorter length of postoperative hospital stay. These results are in accordance with previous systematic revision series^15,17,18^.

In general, ERAS guidelines for elective colon surgery include 21 items^19^; in our series, we tailored the elective ERAS items for colorectal surgery to twenty adapted items for patients with left colon perforation, as prior publications for emergency surgery did^8,10^.

In 2011 Gustafson *et al*.^20^ demonstrated that compliance is essential in the implementation of the elective ERAS protocol. Thus, compliance greater than 70% results in benefits for patients; in contrast, compliance lower than 50% does not result in any benefit for patients. In the series by Loshiriwat^18^ with patients with intra-abdominal infection, compliance was 50%; in our series, global compliance was 62%, probably due to the patients being more homogeneous and selected. Regarding the different periods of the ERAS protocol, compliance greater than 70% was observed in preoperative items 5 and 6 and in postoperative items 5 and 7; only intraoperative items 2 and 7 showed compliance greater than 60%. In this last period, some issues must be considered. In this sense, the role of the laparoscopic approach in emergency colon surgery is debatable and must be carefully taken into consideration. Thus, Rea *et al*.^21^, in a study on emergency laparoscopic resection for acute diverticulitis in 67,645 patients, found the laparoscopic approach was successfully used only in 3.9% of the patients, with a 55% conversion rate. Furthermore, some studies on laparoscopic lavage and drainage of septic focus showed controversial results^22,23^. While two consensus documents^24,25^ state that laparoscopic lavage is not recommended in Hinchey IV, is safe in case of Hinchey III but it is not considered the preferred choice (Recommendation 1 A). These same papers concluded that “laparoscopic sigmoid resection is feasible and safe in selected patients, hemodynamically stable, without significant comorbidities and with onset peritonitis <12-24 h, only in specific advanced laparoscopic colorectal expertise is available (Recommendation 2 C). Considering these recommendations and our results (with 13.7% compliance in laparoscopic colectomy and 10.3% in laparoscopic lavage and drainage of septic focus) we assume that the laparoscopic approach is not recommended as an ERAS item for emergency colon surgery.

Successful implementation of these recommendations is challenging based on their compliance due to existing barriers. Arroyo *et al*.^26^ observed that adherence to the protocol was greater in smaller and less complex hospitals, thus revealing a direct relationship with a shorter hospital stay. According to these results, we assume that the size of our hospitals and previous ERAS experience were essential during the implementation on emergency settings.

Our study has several limitations. First, the retrospective control group is subject to an inherent selection bias. However, it was not subject to selection and, ethically, we cannot currently consider a control group without enhanced recovery care. Secondly, this study has a relatively small sample size, since recruitment of this type of patients is not frequent. To the best of our knowledge, this is the first published study regarding the applicability of a specific ERAS protocol in the management of patients with left colon perforation requiring emergency colon surgery. This work can be used as a pilot study for future randomized studies.

In conclusion, the results of our study show that the implementation of ERAS guidelines in patients with peritonitis secondary to left colon perforation and in patients with a low to moderate risk of mortality according to the PSS is feasible and contributes to better management and patient outcomes. Previous experience with the management of elective surgical patients using these ERAS guidelines was essential to obtain these results. However, prospective and randomised studies are required to support and clarify these results.
