# Publisher Correction: Feasibility and effects of enhanced recovery vs. conventional care after emergency colon surgery for patients with left colon perforation

**DOI:** 10.1038/s41598-020-68285-8

**Published:** 2020-07-08

**Authors:** X. Viñas, E. Macarulla, C. Brugiotti, J. M. Ramirez, A. Pedregosa, S. Sanchez, J. Camps, A. Arroyo

**Affiliations:** 1 Hospital General de Igualada. Servicio de Cirugía General y del Aparato Digestivo. Igualada, 08700 Barcelona, Spain; 2 Hospital General de Inca. Servicio de Cirugía General y del Aparato Digestivo. Inca, 0730 Mallorca, Spain; 3 Hospital General Universitario de Elche. Servicio de Cirugía General y del Aparato Digestivo. Elche, 03203 Alicante, Spain; 4 Hospital Clínico Universitario Lozano Blesa. Zaragoza. Servicio de Cirugía General y del Aparato Digestivo, Zaragoza, 50009 Spain; 5 Hospital General de Igualada. Servicio de Anestesiología y Reanimación. Igualada, 08700 Barcelona, Spain

Correction to: *Scientific Reports* 10.1038/s41598-020-64242-7, published online 30 April 2020

The Article contains errors in Table 3 where the ‘CC group’ and ‘Statistical significance’ values for ‘PSS score’ are missing.

The correct values should read 6.9 ± 1.04 and N/S respectively.

